# The Evidence That Brain Cancers Could Be Effectively Treated with In-Home Radiofrequency Waves

**DOI:** 10.3390/cancers17162665

**Published:** 2025-08-15

**Authors:** Gary W. Arendash

**Affiliations:** RF Longevity SE, Phoenix, AZ 85022, USA; arendash@RFlongevity.com

**Keywords:** brain cancer, gliomas, treatment, non-thermal radiofrequency waves, meningeal lymph flow, immune trafficking, cytokines, rebalancing

## Abstract

There is currently no effective therapeutic capable of arresting or inducing regression of primary or metastatic brain cancers. This article presents both pre-clinical and clinical studies supportive that a new bioengineered technology could induce regression and/or elimination of primary and metastatic brain cancers through three disease-modifying mechanisms. Transcranial Radiofrequency Wave Treatment (TRFT) is non-thermal, non-invasive and self-administered in-home to safely provide radiofrequency waves to the entire human brain. Since TRFT has already been shown to stop and reverse the cognitive decline of Alzheimer’s Disease in small studies, evidence is provided that three key mechanisms of TRFT action, alone or in synergy, could effectively treat brain cancers: (1) enhancement of brain meningeal lymph flow to increase immune trafficking between the brain cancer and cervical lymph nodes, resulting in a robust immune attack on the brain cancer; (2) rebalancing of the immune system’s cytokines within the brain or brain cancer environment to decrease inflammation therein and thus make for an inhospitable environment for brain cancer growth; (3) direct anti-proliferation/antigrowth affects within the brain tumor microenvironment. Importantly, these mechanisms of TRFT action could be effective against both visualized brain tumors and those that are yet too small to be identified through brain imaging. The existing animal and human clinical evidence presented in this perspective article justifies TRFT to be clinically tested immediately against both primary and metastatic brain cancers as monotherapy or possibly in combination with immune checkpoint inhibitors.

## Simple Summary

As there are currently no effective therapeutic interventions against brain cancers for long-term patient survival, a new bioengineered technology is presented wherein both pre-clinical and clinical studies indicate it is safe and would be effective against both primary and metastatic brain cancers. Non-thermal Transcranial Radiofrequency Wave Treatment (TRFT) is self-administered in-home and should have three key mechanisms of actions against brain cancers. The case is made for immediate clinical trials of TRFT in brain cancer patients to induce tumor regression/elimination and long-term patient survival.

## 1. Introduction

Brain tumors, both primary and metastatic tumors, are among the most dreaded and deadly forms of cancer, having few treatment options and a poor prognosis [1]. Although primary brain tumors include Chordomas, Ependymomas, Schwannomas, and pituitary tumors, the most prevalent are glial cell cancers (called “gliomas”) such as glioblastomas (GBs), astrocytomas, oligodendrogliomas, and oligoastrocytomas. Collectively, malignant gliomas are responsible for around 70% of all primary brain cancers, with more than 10,000 individuals dying of glioblastomas in the United States every year according to the National Brain Tumor Society.

The World Health Organization has designed four classes of gliomas. Grade I (astrocytomas) are usually circumscribed with clear boarders and low grade. Grade II (diffuse astrocytomas), Grade III (anaplastic astrocytomas) and Grade IV (glioblastomas) denote progressive stages of malignancy and brain infiltration. As the most aggressive Grade IV subtype, GBs are usually characterized by iso-citrate dehydrogenase (IDH) “wild-type” status (Primary GBs), which is further distinguished from Grades I–III by molecularly based epidermal growth factor receptor (EGFT) amplification, telomerase reverse transcriptase (TERT) protomer mutations, and chromosomal abnormalities [2,3]. Collectively, these attributes produce a highly invasive Grade IV GB tumor that is most resistant to conventional therapies. By contrast, IDH “mutant” gliomas are commonly found in lower-grade gliomas, as well as secondary GBs. In being critical for discriminating between glioma sub-types, IDH1 and IDH2 mutations are essentially absent in primary GBs.

**Figure 1:**
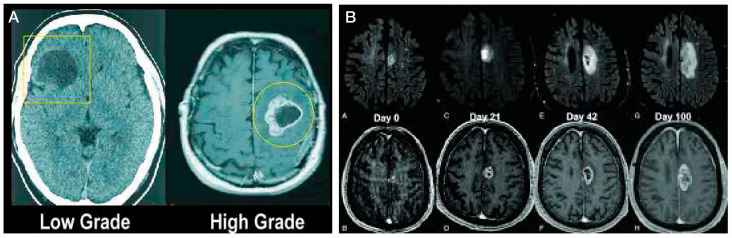
(**A**) Low grade cerebral gliomas typically show only a low-density area (dark area) in MRI scans, whereas high grade gliomas usually show more contrast enhancement (white on the outside and necrosis in the middle (looks black on the MRI). (**B**) Rapid glioblastoma cancer progression in size over a 100-day period.

Cerebral gliomas may be either low grade (Grades I–II) or high grade (Grades III–IV) (Figure 1A), with higher grade tumors often growing rapidly over the course of only a few months (Figure 1B). They are characterized by microvascular development, with tumor arteries providing a plentiful blood source to aid the rapid proliferation of tumor cells [4]. Glioblastomas are further characterized by areas of necrosis, which typically are encircled by cancer cells. Because of glioblastoma tumor rapid expansion, these necrotic areas have localized oxygen deficiency as a consequence of the tumor’s rapid expansion.

In moving beyond histologic grading alone, Verhaak et al. [5] have further defined GBs into four sub-types (Preneural, Neural, Classical, and Mesenchymal) based on molecular classification. Preneural GBs are enriched in platelet-derived growth factor receptor alpha (PDGFR-ɑ) and IDH1 mutations, Neural GBs cells have similarities in gene expression with normal neurons, and Classical GBs are characterized as having EGFR and chromosome 7 amplification, chromosome 10 loss, and activation of Notch signaling pathways. In being the most aggressive subtype, Mesenchymal GBs are categorized by extensive necrosis, upregulation of angiogenesis genes (VEFG-A, VEGF-B), and frequent deletions within the tumor suppressor genes tumor protein 53, PTEN, and NF1. These molecular-based sub-types have different tumor progressions and survival outcomes, which make them important for determining the best treatment options.

In addition to a molecular-based refinement of GB classification, measurement of DNA methylation within tumors provides further GB subtyping information. More specifically, six methylation clusters (M1–M6) have been found, with each cluster having a distinct prognosis indicator. For example, the tumor presence of cluster M5 involves hypermethylation and frequent IDH1 mutations, which are linked to less aggressive tumor proliferation and better survival. However, tumor presence of cluster M6 involves hypomethylation and a predominance of IDH1 wild-type cells, which translates to more aggressive tumor proliferation and a poorer prognosis. *Thus, the integration of histologic grading with molecular/genetic alternations and DNA methylation patterns provides a refined and more comprehensive stratification of gliomas from which a more accurate prognosis and more informed therapeutic decisions can be made*.

Survival/mortality rates for glioblastoma (Grade IV) patients have been virtually unchanged for decades, with a five-year survival rate of around 5% [6] and relapse being inevitable [6,7,8]. Following recurrence, GB patients have an overall survival of between 6 and 9 months—less with increased age [7,9,10]. There are no known GB risk factors other than rare cases of genetic susceptibility and radiation exposure at high doses [11]. Glioblastomas in particular have a strong propensity to spread to other brain areas, but do not spread to other parts of the body [6,12,13]. Nonetheless, metastatic brain cancers spreading to the brain from other locations in the body (e.g., from lungs, breast, skin melanoma, colon) remain the most frequently occurring brain cancers [14]. In this regard, metastatic lung cancer and melanoma both have a marked predilection for spreading to the brain [15]. Indeed, brain metastasis from peripheral tissues/organs exhibits more than ten times the incidence of primary brain tumors [16] and a striking 20% of cancer patients will develop brain metastasis [17]. The incidence of brain metastasis has been steadily increasing and, as with primary brain tumors (e.g., gliomas), the prognosis for brain metastasis patients remains poor.

Cancer “metastasis” to the brain results from dissemination of tumor cells from their primary site (e.g., lung, melanoma) to enter the blood circulation and become the circulating tumor cells. After spreading throughout the circulatory system to the brain microvasculature, tumor cells penetrate the blood–brain barrier (BBB) and locate within brain tissue, which often provides a favorable environment for their growth [18]. Parenthetically, discriminating brain metastatic lesions from single ring glioblastomas remain challenging.

Although long-term survival rates among patients with malignant primary or metastatic cancers remain low, therapeutic advances to slow or arrest these cancers are being made and represent meaningful progress. Some of the treatment options for brain cancer patients are surgical resection, radiosurgery, chemotherapy, targeted therapy, immunotherapy, or a combination therein [19]. The current standard of care (SOC) includes maximal safe resection and radiotherapy, with concurrent and/or adjuvant drugs such as Temozolomide (TMZ). This SOC results in an improved overall survival from 12.1 to 14.6 months and an increase in the survival rate at two years from 10% to 27% [6]. Regarding glioblastomas, their highly invasive nature and dysregulation of many biochemical pathways typically does not allow for complete resection and limited radiation success [1,11]. Chemotherapies have been shown to inhibit tumor growth by damaging the DNA of tumor cells; nonetheless, many patients develop a resistance to these drugs. As for targeted therapy, drugs targeting key pathogenic molecules such as vascular endothelial growth factor (VEGF) and EGFR have not provided satisfactory outcomes [20]. Most recent, research focus has been on immunotherapies such as checkpoint inhibitors and dendritic cell vaccines. Such immunotherapies aim to enhance the body’s immune response to resist the tumor [21]. Despite gliomas being scientifically identified a century ago, there have only been five drugs and one device ever approved by FDA for treatment of glioblastoma since then. Nonetheless, modest clinical advances have been made and continue to be made through a large number of clinical trials that are ongoing.

Comparing the overall survival rates provided by various treatment options (e.g., surgical resection, radiosurgery, chemotherapy, targeted therapy, immunotherapy) separately is difficult in view of patients having: (1) different types of gliomas or brain metastases; (2) different tumor grades; (3) different tumor locations; (4) different progression at the start of therapy; (5) different molecular/DNA methylation patterns; (6) treatment involving multiple options among those listed above. As such, it would be nearly impossible to compare overall survival (OS) or progression-free survival (PFS) separately between the various treatment options for gliomas. Rather, meta-analyses and systemic reviews have focused on various combinations of treatment options to attempt determination of the most effective treatment for gliomas. Unfortunately, no consensus has yet been reached, with clinical outcomes from various treatment combinations in glioblastoma remaining particularly inconsistent. However, a very recent umbrella review of 68 meta-analyses [22] has provided some insight into GB treatment efficacy by focusing on higher quality treatment studies. The authors conclude that chemoradiation with or without targeted therapy, and targeted therapy with or without chemotherapy were associated with improved OS and PFS. Unfortunately, such combinations appeared to increase adverse events. Despite significant advancements in understanding GBM sub-types and pathogenesis, more effective treatments have remained elusive because of tumor heterogeneity, adaptability, and the need for interactions with multiple cell types within the tumor microenvironment.

The present perspectives paper first reviews current therapies against brain cancers with a focus on glioblastomas, then justifies the brain’s meningeal lymphatic and cytokine systems as excellent targets to treat brain cancers. Animal studies are then presented showing the ability of the cytokine VEGF to enhance the immune response to brain tumors by increasing lymph flow thorough the brain’s meningeal lymphatic vessels (mLVs). Finally, this paper proposes Transcranial Radiofrequency Wave Treatment (TRFT) as a new and promising non-pharmacologic intervention against brain cancers because it: (1) has potentially “disease-modifying” mechanisms through modulation of VEGF/mLVs and re-balancing the immune system; (2) can target both visualized and small (unseen) growths within the human brain; (3) has already been shown to clinically benefit Alzheimer’s Disease patients in several studies. Through presentation of relevant pre-clinical and clinical studies, the case is made for immediate clinical trials of TRFT in patients bearing either low- or high-grade malignant gliomas or metastatic brain cancers.

## 2. Current Therapeutic Interventions Against Brain Cancers

Current interventions against brain cancers are either non-pharmacologic (surgical resection, radiosurgery, ultrasound ablation, tumor-treating electric fields) or systemic/pharmacologic (chemotherapy, targeted therapy, immunotherapy). For systemic therapies in general, impermeability of the blood–brain barrier (BBB) limits their transport into the brain/tumor. As such, systemic therapies are less effective in treating brain cancers than for treating other types of cancers [18]. Typically, a combination of several interventions is utilized [23,24,25]. Indeed, combination therapy with surgical resection, radiosurgery, and chemotherapy continue to be the mainstay for glioblastoma patients, although their combined efficacy is limited and the survival of glioblastoma patients continues to be short [1]. Given the heterogeneity of brain metastasis, there is currently no consensus regarding the sequence of treatment and combination therapy is often disappointing [18].

### 2.1. Non-Pharmacologic Interventions

Surgical Resection: Removing brain cancer cells without causing damage to critical brain regions is a daunting task for surgeons. If they are too aggressive in brain cancer cell removal, they could easily cause cognitive impairment and/or neurologic deficits. The surgical removal of both primary tumors and tumors resulting from brain metastasis can reduce symptoms and moderately prolong life expectancy [26,27]. However, the unclear boundaries of a brain tumor often result in residual tumor cells surviving after surgery, bringing about tumor reemergence [1]. To optimize efficacy of resection and minimize residual tumor cells, a variety of accompanying implements are employed. Chief among these implements are better visualization of the tumor using 5-aminoevulinicacid (5-ALA) and intraoperative use of MRI for improved definition of the tumor [28,29,30]. Indeed, the combination of these two implements with surgical resection resulted in a 95% resection volume [31,32].

Radiosurgery: Following surgical resection, postoperative radiotherapy is used to eliminate minor areas of unresectable tumor tissue and increase survival in glioblastoma patients [6,33]. In brain metastasis patients as well, radiosurgery is a cornerstone (along with surgical ablation) of disease management. Radiosurgery exerts therapeutic effects by delivering high doses of radiofrequency “thermal radiation” to brain tumor tissue, causing cell death in as little as 30 seconds once cell temperatures reach 50 °C. Such thermal-based RF approaches to solid cancer treatment have the drawback that they have little ability to spare normal structures in the treatment zone. Nonetheless, with diagnostic MRI, radiation can be limited to a specific location [34]. With its low rate of complications and less deterioration of neurocognitive functions [18], radiosurgery is generally started 4 to 6 weeks post-ablation surgery and/or in combination with chemotherapy. Unfortunately, tumor recurrence remains inevitable because of tumor environmental resistance to radiation [35].

Ultrasound ablation: There are two forms of focused ultrasound (FUS) being investigated against brain tumors: high- and low-intensity FUS. High intensity FUS produces heat by causing the vibration of molecules within the tissue. Localized brain temperature is quickly elevated to over 55 °C by the absorbed energy, which causes DNA fragmentation, protein denaturation, and coagulative necrosis [36,37]. Clinical trials with high-intensity FUS have thus far been limited to Phase I and case studies to achieve glioblastoma ablation, with no therapeutic results published. In sharp contrast, low intensity FUS uses lower energy pulsed waves (around 500 kHz) to cause mechanical perturbation and acoustic cavitation of systemically injected microbubbles within the targeted brain area. This “oscillation” of microbubbles temporarily increases permeability of the BBB within the targeted region to increase brain penetration of therapeutic agents such as TMZ. Only a couple of small studies have been reported with low-intensity FUS in glioblastoma patients, with results showing improved survival of a few months [38,39].

Tumor-Treating Electric Fields: A relatively new approach specifically for brain glioblastomas is the use of Tumor Treating Fields (TTFs), which are alternating electric fields (100–400 kHz) generated by electrical current running through a gridded mat placed on the bald head of GB patients for a long 18 h a day. TTFs, which act in general by impeding division of cancer cells, add a modest number of months to survival of glioblastoma patients. More specific, TTFs inhibit mitosis, cause chromosome mis-segregation, and disrupt DNA repair in GB cells [40,41,42,43,44]. In recent years, TTFs has become a complementary treatment strategy, which is now part of the standard of care for GB treatment [45,46,47,48]. The approval by FDA of TTFs to treat GB was grounded on results from a clinical trial (NCT00379470) showing evidence that TTF monotherapy offered similar efficacy compared to chemotherapy in patients with recurrent GB—this, with less toxicity, a reduction in serious adverse events, and a better quality of life [49]. The addition of TTFs to standard of care therapy improved overall survival of GB patients by nearly five months [50,51,52]. TTFs are usually applied within 6 weeks after the end of radiosurgery/chemotherapy [48,50]. Despite the apparent benefits associated with TTFs for GB treatment, its clinical use is still quite restricted. Some clinicians maintain skepticism regarding the use of TTFs because of the lack of effect in some patients and because of the extraordinarily long daily treatment periods (18 h per day) on a balded head being required for modest benefit, both of which limit its utility.

### 2.2. Pharmacologic Interventions

As is currently the case for non-pharmacologic therapeutics, systemic/pharmacologic interventions against brain cancers can provide some months of additional living. Despite intense research efforts and many clinical trials, no pharmacological intervention has been demonstrated to alter the course of glioblastoma disease [53,54]. Similarly, recent trials involving “systemic” drugs to complement radiotherapy in patients with brain metastasis have indicated that their efficacy for intracranial metastatic cancers is still grim and systemic treatment options can provide only limited benefits [18,55]. Systemic drugs have thus far been ineffective in clinical trials in part because they do not get into the brain tumor site very well due to their having to cross both the blood-brain and brain-tumor barriers. Thus, systemic drugs are often in too low a concentration at the tumor site(s) to provide treatment efficacy. To increase penetration through the blood–brain barrier and brain-tumor barrier, nanoparticle-mediated delivery of chemotherapeutic drugs to glioblastomas and brain metastasis patients is presently being investigated. Current available systemic therapies for brain cancer patients include chemotherapy, targeted therapy, and immunotherapy, as discussed below.

Chemotherapy: The efficacy of chemotherapeutic drugs lies in their ability to damage tumor cell DNA, leading to either cancer cell death or the cessation of their proliferation. Three chemotherapy drugs have been approved by FDA for treatment of glioblastomas. Since usage of two of these drugs (Carmustine and Lomustine) is usually halted during treatment due to liver and kidney toxicity [56], Temozolomide (TMZ) stands as the primary FDA-sanctioned drug for glioblastoma treatment—it is also the primary chemotherapy for metastatic brain cancer. TMZ is a mono-alkylating agent that is cytotoxic to brain cancer cells by modifying tumor cell DNA through methylation, obstructing DNA replication, and inducing apoptosis [57]. Although it is a cornerstone of glioblastoma treatment, TMZ’s effectiveness is limited by the aforementioned blood–brain and blood–tumor barriers, as well as inherent or acquired glioblastoma resistance [58,59,60,61]. TMZ, when given alongside radiotherapy, improves average survival for people with high grade brain tumors, compared to those who only have radiotherapy—thus its global use as the main chemotherapeutic drug against glioblastomas.

Targeted Therapy: There are many drug-based therapies that have been shown to interfere with different signaling pathways involved in glioblastoma tumorigenesis, pathophysiology, and treatment resistance acquisition (e.g., the pRB/CDK4/RB1/P16ink4 pathway, the TP53/MDM2/P14arf pathway, the PI3k/Akt-PTEN pathway, the RAS/RAF/MEK pathway, PARP, and EGFRs) [11]. Unfortunately, clinical trials involving such drugs have thus far not resulted in benefits against glioblastomas. For lung cancer brain metastasis, most targeted therapies are those targeting driver mutations of EGFR, ALK, ROS1, RET, and MET, as well as driver mutation-associated signaling pathways, such as RAS/RAF, PI3K/AKT/mTOR, WNT/β-catenin, and JAK/STAT [18]. As with glioblastomas, the efficacy of such systemic/targeted therapies for intracranial metastases remains low and treatment options for brain metastasis remain limited. See Obrador et al. [11] and Lu et al. [18] for extensive reviews of the many targeted drug therapies that have been or are currently being investigated against glioblastoma and metastatic brain cancer.

Immunotherapy: Ideally, the body’s immune system would be called in to attack, kill, and/or contain brain cancers. Unfortunately, the immune response to the presence of brain cancers such as gliomas is minimal and ineffective, as detailed in Section 4. This is due firstly to an insufficiency of lymphatic vessels/flow within the brain parenchyma through which to transport specific memory T cells from the glioma to cervical lymph nodes for signal amplification. Secondly, the immune response to cancers in the brain is inherently small and insufficient for inducing arrest or regression of brain cancers, most notably gliomas. This later issue is important since, if the immune system could respond more vigorously to the brain tumor, there is reasonably expectation that an arresting of tumor growth or actual brain tumor regression may occur. In an attempt to enhance the immune response against glioblastomas and thereby increase patient survival, several immunotherapeutic approaches have been utilized. While immunotherapies have revolutionized treatment for other cancers, similar benefits have not been achieved in brain cancer patients due to the tumor’s immunosuppressive local environment. Specifically, brain cancers have an “immunologically privileged” nature characterized by cytotoxic T cell exhaustion, limited lymphocytic infiltration, and an abundance of immune-inhibitory molecules such as IL-10 and TGFβ [11]. Listed below are some current immunotherapeutic drugs utilized against primary and/or metastatic brain cancers:

### 2.3. Immune Checkpoint Inhibitors

Cytotoxic T lymphocyte antigen 4 (CTLA-4) and programmed cell death 1 (PD-1), also known as immune checkpoints, are co-inhibitory receptors expressed on the T cells surface to promote immunotolerance. Glioblastoma cells (and other cancer cells) overexpress programmed cell death ligand 1 (PD-L1) which, upon interaction with PD-1, inhibits T cell proliferation and suppresses the CD4+ and CD8+ response [62]. An assortment of “checkpoint inhibitors” have been approved by FDA to overcome these immune checkpoints for the treatment of various types of cancer [63]. The most widely used monoclonal antibodies for blocking such immune checkpoints are anti-PD-1 and anti-CTLA-4. These agents target PD-1 (nivolumab, pembrolizumab, and cemiplimab), CTLA-4 (ipilimumab), or PD-1’s associated programmed cell death ligand (PD-L1) (avelumab, durvalumab, and atezolimumab) on T-cells. Although such antibodies have indeed been found successful to differing extents in several mouse models of glioma [64], several recent phase III clinical trials in glioblastoma patients have resulted in none of them significantly increasing overall survival of glioblastoma patients [11,65,66,67]. Similarly, in lung cancer patients with brain metastasis, immune check point inhibitors alone have minimal benefits, so combination of immune checkpoint inhibitors with radiotherapy or/and chemotherapy are currently needed [68,69]. In that regard, a meta-analysis of 19 studies in lung cancer patients with brain metastasis concluded that a combination of immune checkpoint inhibitors and radiation results in modestly increased overall survival [70].

### 2.4. Dendritic Cell Immunotherapy

For dendritic cell immunotherapy, the patient’s immature immune cells are coaxed into growing into dendritic cells, which may then boost the immune system’s attack on a given brain cancer. Once these cells have been produced, they are modified to train the patient’s own immune T cells to attack certain proteins, or antigens, on the surface of tumor cells in the brain that are not on the surface of normal cells. More specifically, dendritic cell vaccine preparation involves the isolation of CD-14 positive monocytes from the patient, loading of tumor antigens into the immature dendritic cells, treatment of the dendritic cells with various cytokines (e.g., GM-CSF and IL-4) to induce maturity, and finally re-injection of the dendritic cell vaccine into glioblastoma patients [71,72,73]. Results of Phase II/III trials with dendritic cell vaccines in glioblastoma patients have been variable, with some studies showing no effect on survivability [74,75] and others showing enhanced survival of a few months [76,77].

### 2.5. Anti-VEGF Monoclonal Antibody (Bevacizumab)

Targeting tumor angiogenesis has been an attractive therapeutic modality in glioblastoma and other brain cancers. One of the best-known angiogenesis stimulators in glioblastoma progression is VEGF [78], which is upregulated in GB tumors. As an antibody targeting VEGF, Bevacizumab hinders the process of angiogenesis by counteracting VEGF and impeding its adherence to VEGF receptors [79]. However, trying to target VEGF for glioblastoma faces substantial obstacles, most notably regarding resistance to treatment. For example, tumor cells can combat anti-VEGF therapies by enhancing other angiogenic factors. (such as basic FGF) or activating alternative pathways such as Ang-2 signaling [1]. Clinical trials evaluating Bevacizumab as monotherapy or in combination with lomustine or TMZ have not resulted in improved overall survival in glioblastoma patients [80,81,82,83,84,85]. As well, lack of benefits was also observed in clinical trials where Bevacizumab was combined with temsirolimus, erlotinib, sorafenib, or tandutinib in recurrent glioblastomas [86,87,88,89]. Given the aforementioned resistance to anti-VEGF treatment, as well as insufficient drug delivery to brain tumors because of the blood–brain barrier, it is not surprising that clinical trials with Bevacizumab to treat gliomas have been unsuccessful.

Combination Pharmaceutical and Non-Pharmaceutical Interventions: Combining specific treatment modalities and in what sequence could be critical for attaining benefits in brain cancer patients. As such, many current “combination” clinical trials have evaluated, or are currently evaluating, the best combinations of surgical ablation, radiosurgery, chemotherapy, targeted therapy, and/or immunotherapy [19]. For example, several retrospective studies have demonstrated benefits of combination radiotherapy and immunotherapy in patients with brain metastasis of lung cancer [90]. In several combinations investigated in postoperative glioblastoma patients, it was unclear whether the combination of chemotherapy with radiosurgery or immune checkpoint inhibition had any effects on overall survival [91]. TMZ, when given alongside radiotherapy, modestly improved average survival for people with high grade brain tumors, compared to those who only have radiotherapy [11]. In an encouraging clinical trial involving oral Vorasidenib (an inhibitor of mutant IDH1 and IDH2 enzymes) to patients bearing IDH1- or IDH2-mutant low-grade glioma who had previous surgery for tumor removal, progression-free survival to the next intervention was significantly increased to 27.7 months versus 11.1 months for placebo [92]. Many additional combination trials are in process, including a clinical trial of combination Bevadizumab and radiosurgery (NCT05611645).

To summarize, current clinical interventions against primary and metastatic brain cancers can provide a limited benefit for additional living, but unfortunately with no long-term benefits. Novel therapeutic targets and associated interventions are desperately needed wherein relevant pre-clinical/and clinical studies support their potential to induce regression and/or elimination of brain cancers—especially at cancer locations in the brain that are not yet visible via imaging techniques. In view of supportive pre-clinical and clinical studies, the present paper proposes use of a new bioengineered technology—non-thermal Transcranial Radiofrequency Wave Treatment (TRFT)—as a novel and safe in-home approach to potentially treat all types of primary and metastatic brain cancers, resulting in long-term survival.

## 3. A New Bioengineered Intervention Against Brain Cancers: Transcranial Non-Thermal Radiofrequency Wave Treatment

In view of the preceding Section on current therapeutics, there is a huge unmet need for therapeutics against brain cancers that can provide a robust attack resulting in tumor regression and long-term patient survival. This section provides an introduction to “non-thermal” Transcranial Radiofrequency Wave Treatment (TRFT) as a new bioengineered technology that should provide an effective multiple-pronged, non-invasive intervention against potentially all forms of brain cancer. The history of radiofrequency (RF) waves and brain cancer is first presented, followed by a brief summary of pre-clinical RF studies in AD mice. Then a device is described (MemorEM) that safely delivers full brain RF waves to humans. Clinical studies with this device will then be presented that demonstrate therapeutic use of TRFT to stop and reverse a major neurologic disease of against—Alzheimer’s Disease—and through multiple mechanisms. Finally, in succeeding sections, two recently discovered mechanisms of TRFT action will be introduced that are shared by both AD and brain cancers.

**Figure 2:**
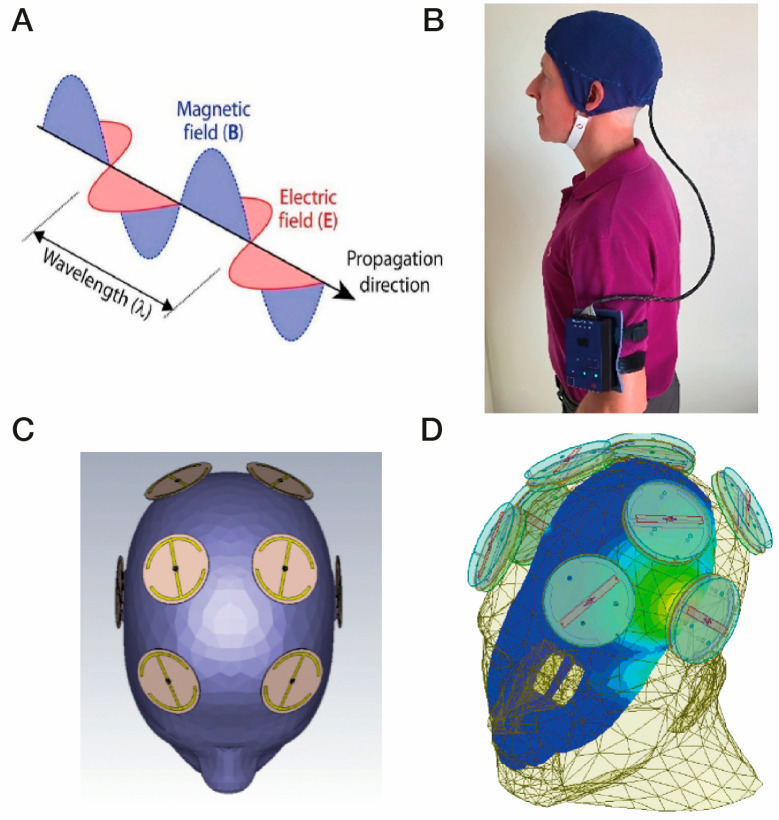
(**A**) Electromagnetic/radiofrequency waves each comprise an electric and a magnetic wave at right angles to one another. (**B**) A MemorEM device, consisting of a battery/RF generation box worn on the arm that is connected to a head cap, the device allowing near complete mobility during in-home treatments. (**C**) Location of the eight electromagnetic/radiofrequency wave emitters between the two-layered head cap. (**D**) An FDTD (ANSYS) brain simulation showing a single emitter being “ON” (emitter located above the left ear). The simulation shows the emitter’s electric field penetration and distribution into the temporal lobe at a power level of 1.6 W/kg. Figure 2A–C are adapted from Arendash et al. [102].

*RF Waves and Brain Cancer*. Radiofrequency waves are at the lower end of electromagnetic wave frequencies, ranging between 300 MHz through 3 GHz. Each RF wave consists of linked sinusoidal electric and magnetic “waves”, generating an electric field, magnetic field, and direction of wave propagation all aligned perpendicular to each other (Figure 2A). These RF waves radiate away from their emitter, never to return, which contrasts with the circulating magnetic fields of both Transcranial Magnetic Stimulation and pulsed electromagnetic stimulation. Early generation cell phones produced RF frequencies primarily around 900 MHz and 1800 MHz for transmission and reception of phone messages. Because RF waves in that frequency range easily penetrate the human cranium and underlying brain areas, there was some concern in the early 2000s from epidemiologic studies done mostly by a single research group that cell phone use doubles risk of brain cancers from 0.5% to 1% [93,94]. Since then, however, large and well-designed human epidemiologic studies have concluded time and time again that long-term exposure to RF fields of around 900 MHz have no negative impact on health, particularly on incidence of brain tumors. For example, the large INTERPHONE Study [95] found no increased risk of brain cancer over a 10+ year period of cell phone use. Indeed, that 13-nation study found a significantly lower risk of both gliomas and meningiomas in regular users compared with people who were not users—a “protective” effect of RF waves against brain cancers. Similarly, in a huge study of 358,000 cell phone users in Denmark, no risk or a reduced risk of brain tumors was found among regular male cell phone users compared to non-users—regular use was associated with an odds ratio (OR) of 0.79 for meningioma and 0.81 for glioma [96]. Finally, a huge review by Karpitis et al. [97] involving 63 papers concluded there was moderate certainty that RF waves from cell phones do not increase risk of glioma, meningioma, acoustic neuroma, or pediatric brain tumors. Based on such studies, major health organizations (e.g., NCI, FCC, NIEHS, CDC) have concluded there are no health problems (including brain cancers) that have been linked to RF wave exposure between 900 and 1800 MHz—indeed, just the opposite has been found in both animals and humans.

*Pre-Clinical Studies*. Between 2010 and 2012, results of multiple pre-clinical studies in Alzheimer’s transgenic mice exposed to non-thermal RF waves at 918 MHz frequency and 1.6 W/kg power level were reported [98,99,100,101]. These AD mice bear the human genetic mutation(s) that cause synthesis and aggregation of the toxic protein amyloid-β (Aβ) in their brains. Over the course of months to a year, they develop the Aβ neuropathology and cognitive impairment that has served as the premiere animal model(s) for AD. These pre-clinical studies demonstrated a surprising ability of daily RF wave treatment against brain Aβ aggregation and memory loss. Specifically, if RF waves were given in young adulthood, it prevented both Aβ aggregation and onset of cognitive impairment [98]. If given in older age, RF waves both reverses brain A**β** and cognitive impairment [99]. A second mechanism of action that was discovered and probably contributory to the cognitive benefits of RF waves is “mitochondrial enhancement” to induce more brain energy production by enhancing mitochondrial Complex IV activity in neurons [101].

*The MemorEM Device*. The above cognitive benefits and two mechanisms of RF wave action justified translation into clinical trials of this technology against human Alzheimer’s Disease. My colleagues and I thus developed a first-of-its-kind head device for administering RF waves to the entire human brain. This bioengineered device, called the “MemorEM”, comprises a head cap containing eight RF wave emitters between two fabric layers, with each emitted connected to a small RF Generator/battery via a common cable (Figure 2B). The device permits near complete mobility at home, allowing the wearer to perform most home activities while receiving full-brain RF treatment through the eight emitters shown in Figure 2C. Sequential activation (1→8) of these eight emitters occurs 217 times/second (217 Hz), with only one emitter on for any given split second at 915 MHz. Figure 2D displays an FDTD (ANSYS) brain simulation from a single “ON” emitter located above the left ear, showing the emitter’s electric field penetration and distribution into the temporal lobe at a power level of 1.6 W/kg. It can be appreciated that, through all eight emitters, RF treatment is provided to the entire cerebral cortex, sub-cortical areas (e.g., hippocampus), and deep/superficial cerebral blood vessels albeit at decreasing power. In December 2016, the MemorEM device was approved as a “non-significant risk” (NSR) device by the Western Institutional Review Board (WIRB) and in March 2020, the MemorEM device was designated by the FDA as its first “Breakthrough Device” for the treatment of AD.

**Figure 3:**
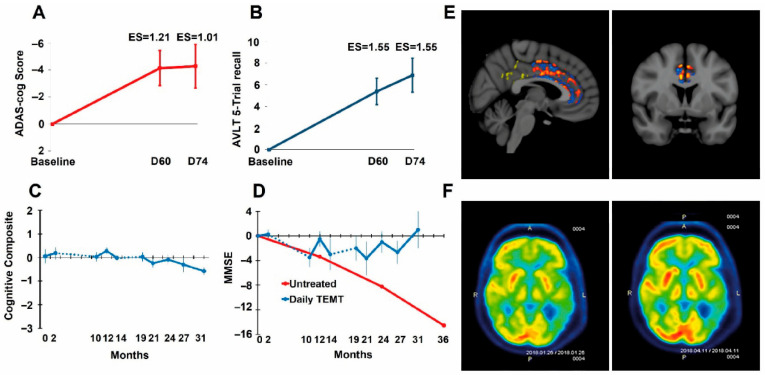
(**A**,**B**) TRFT reverses Alzheimer’s memory impairment in ADAS-cog13 and Rey AVLT 5 Trial Recall. Improvement after 60 days of TRFT was maintained for at least 2 weeks after TRFT; ES = effect size (as Cohen’s d) compared to baseline. An ES greater than 0.8 and 1.20 indicates clinically significant effects that are large and very large, respectively. (**C**) AD patients given TRFT over a period of 2½ years showed no “overall” cognitive decline in a Cognitive Composite score made up of ADAS-cog13, Rey AVLT, MMSE, Digits Forward, Digits Backward, and ADL measures. (**D**) The MMSE component of the Cognitive Composite score showed no significant decline through this extended 2½ year period. The red line and data points are from another study evaluating MMSE longitudinally in a group of “untreated” mild/moderate AD subjects. The dotted lines in (**C**,**D**) indicate the 8-month and 5-month periods of no treatment. (**E**) fMRI images comparing several hundred pixels within the cingulate cortex for pre- vs. post-TRFT (2 month) differences for each pixel with red, orange, and yellow pixels indicative of increased functional connectivity. Typically, only blue pixels (decreased functional connectivity) are present in AD subjects. (**F**) FDG-PET scans from an AD subject taken at baseline (L) and at 2 months into daily TRFT (R) showing cerebral metabolic rate for glucose. Note higher FDG-PET intensity after TRFT throughout the forebrain, as evidenced by more prevalent red/orange areas. Reproduced with permission from Arendash et al. [103,104].

*Clinical Studies**.* Utilizing MemorEM devices, a group of eight mild/moderate AD patients were given twice-daily 1 h treatments of TRFT by their caregivers for 2 months [103]. Compared to baseline, TRFT resulted in highly significant improvements in both ADAS cog 13 and Rey AVLT recall (Figure 3A,B)—a stunning reversal of AD cognitive impairment. Moreover, extending TRFT to 31 months (2½ years) in AD subjects brought about a complete stoppage of cognitive decline over that period [104], as evidenced by no significant cognitive decline in an 8-measure cognitive composite (Figure 3C) and in one example from those eight measures, MMSE scores (Figure 3D). Accompanying these cognitive benefits were TRFT-induced increases in functional connectivity between neurons in MRI scans (Figure 3E) and enhanced FDG- PET scans indicative of increased energy utilization of neurons (Figure 3F) [103]—both of these imaging measures progressively decrease over time in AD patients. All of these TRFT-induced benefits in AD likely involve the two previously mentioned mechanisms of TRFT action—namely, disaggregation of toxic brain proteins (e.g., Aβ) and metabolic/mitochondrial enhancement [98,99,101,103].

Recently, an additional two mechanisms of TRFT action have been identified that target not only AD, but also brain cancers. Pertinent to brain cancers, these two “disease-modifying” mechanisms are: (1) enhancement of brain lymphatic flow to increase immune trafficking/signaling between the brain cancer and cervical lymph nodes (Section 6), and (2) rebalancing of the immune system’s cytokine signaling within the brain tumor and/or the brain in general to decrease brain inflammation therein (Section 8). A third mechanism of TRFT action specific to brain cancers that will be discussed in Section 9 is direct RF wave attack on cells within the tumor’s microenvironment. Thus, a multi-pronged, diversified attack by TRFT on brain cancers is proposed that could be very successful in causing tumor regression and patient long-term survival.

## 4. A First Target of TRFT Against Brain Cancers: Brain Lymphatic Vessels and VEGF

Up until recently, it was believed that there were no functional lymphatic vessels in the brain capable of working in concert with the blood’s immune system to mount a robust attack on brain cancers; this, specifically through lymph node production of immune cells and their transport via blood to the brain tumor location. The only connectivity/communication between the brain and blood immune system was thought to be by brain interstitial fluid drainage into the Cerebrospinal Fluid (CSF), then from CSF into the systemic vascular circulation. However, studies have now described a heretofore unknown (re-discovered) group of lymphatic vessels in the brain, called meningeal lymphatic vessels (mLVs).

**Figure 4:**
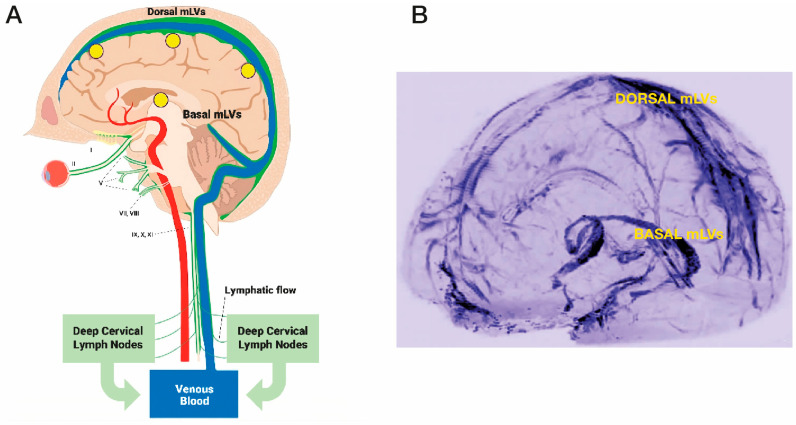
(**A**) Meningeal lymphatic vessels (mLVs), located within the brain’s meninges/dura, consist of both “Dorsal” and “Basal” lymphatic components (green vessels). Note the close parallel relationship of mLVs to venous sinuses (blue) within the brain. Selected cranial nerves are indicated with roman numerals. (**B**) Visualization of Dorsal and Basal meningeal lymphatic vessels in human brain using high-resolution Magnetic Resonance Imaging with Gadobutrol contrast. CSF within mLVs is first transported to cervical lymph nodes and then into the venous circulation. Yellow circles in (**A**) depict the approximate head surface locations on the left side of the head for the four radiofrequency emitters of a MemorEM device (see Figure 2B,C). Figure 2A reproduced from Arendash [106] and Figure 2B adapted from Absinta et al. [107].

**Figure 5:**
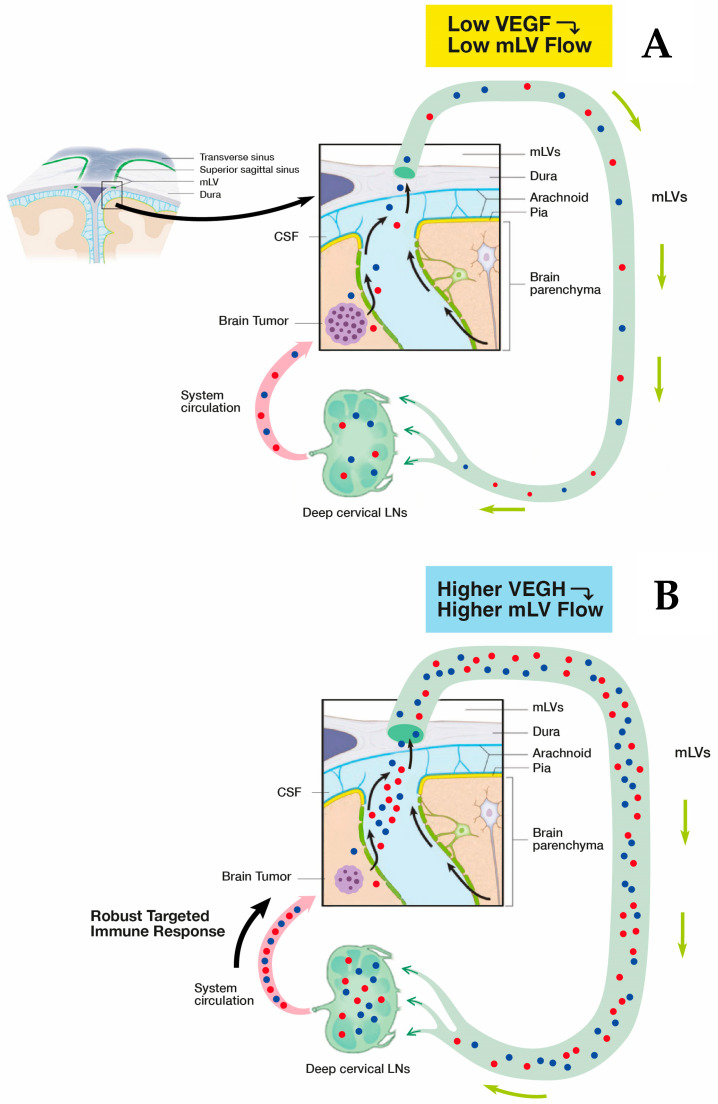
(**A**) The immune system’s response to solid brain tumors (e.g., GB) is weak and ineffective due to minimal drainage of mLV fluid containing specific T cells/dendritic cells (red and blue spheres). This minimal lymphatic drainage via mLVs results in an insufficient presentation of these T cells/dendritic cells to deep cervical lymph nodes and an ensuing minimal immune accentuation of T cells/dendritic cell numbers leaving cervical lymph nodes. The result is an inadequate immune response returning to the brain tumor via the systemic circulation and no effect on tumor viability/growth. (**B**) As the primary modulator of mLV flow, VEGF in higher concentrations around mLVs will increase their flow, resulting in greater transport of specific T cells/dendritic cells to cervical lymph nodes and a consequent much more robust accentuation of their numbers leaving 570 the lymph nodes. Thus, a much stronger specific immune response is presented to the solid brain 571 tumor, which should result in tumor regression.

MLVs are located parallel to dural venous sinuses and are present both dorsally and basally relative to the brain and skull (Figure 4A,B). Collectively, these two lymphatic components account for approximately half of total brain cerebrospinal fluid drainage out of the brain. The “basal” MLVs appear to be primarily a sink for draining toxins (including β-amyloid and p-tau) from the brain. The “dorsal” MLVs seem to be more involved with draining intra-tumor fluid from solid brain cancers into cervical lymph nodes, wherein an immune response can be generated by specific memory T-cells/dendritic cells against a particular brain cancer (Figure 5A) [105]. These memory T-cells/dendritic cells would then travel through the systemic circulation to the brain to provide a specific, immune-based attack on brain tumors (Figure 5A). As such, mLVs can be considered as *“tumor-associated lymphatics”*, linking the brain to peripheral immune responsivity. Unfortunately, the immune system’s response to solid “brain” tumors is weak and ineffective. To a considerable degree, this paltry immune response is due to minimal intra-tumor drainage, which initially goes from the interstitial fluid to mLVs, then to cervical lymph nodes.

**Figure 6:**
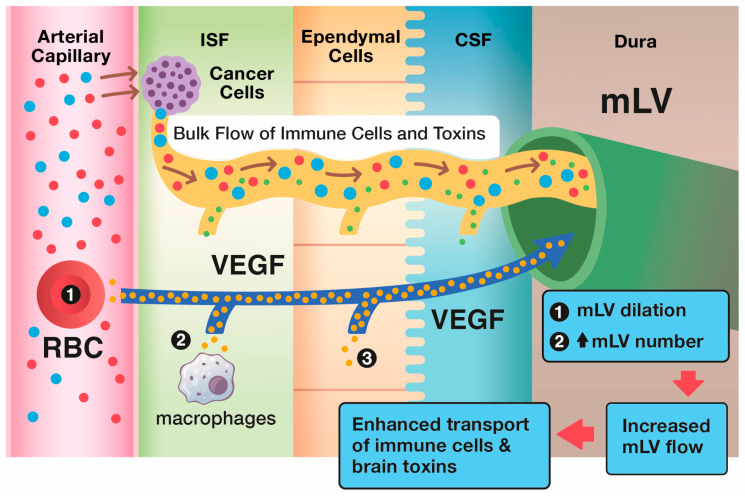
The brain’s primary modulator of mLV flow of CSF out of the brain is the cytokine VEGF. Specifically, VEGF in and around brain mLVs induces their dilation and an increase in mLV numbers (lymphangiogenesis), as shown in the upper blue box. Through both of these processes, VEGF increases CSF flow via mLVs to cervical lymph nodes. As such, an increased number of tumor-specific T-cells and dendritic cells (and drainage of brain toxins) flow from the interstitial brain cancer into mLVs, then to deep cervical lymph nodes to induce a more robust immune response via blood back to the brain cancer. Shown in black circles within their respective tissue types are the brain’s three sources of VEGF: plasma in choroid plexus capillaries, tissue macrophages, and choroid plexus ependymal cells. T-cells (blue), dendritic cells (red), toxins (green), VEGF (yellow).

It seems clear that a new and novel target for therapeutic intervention against brain cancers would be to augment mLV function (i.e., mLV vessel dilation and/or increased mLV numbers) to increase lymph flow from the brain cancer to cervical lymph nodes. This would enhance communication between brain and the immune system to generate a robust, targeted immune response against a given brain tumor (Figure 5 and Figure 6). The primary modulator of mLV flow is the cytokine Vascular Endothelial Growth Factor (VEGF) due it its ability to increase both the diameter and number (lymphangiogenesis) of mLV vessels in the brain. In that regard, there are three sources of VEGF in the brain that can potentially modulate mLV diameter and vessel numbers: (1) within choroid plexus capillaries, VEGF in blood plasma that diffuses to mLVs; (2) secretion by ependymal cells lining the choroid plexus; (3) release from resident macrophages within the choroid plexus interstitial fluid (Figure 6).

Unfortunately, the only current way to increase lymph flow through mLVs is to repeatedly inject VEGF into the CSF or to intracerebrally introduce viral vectors that overexpress mRNA for VEGF. VEGF-C and VEGF-A are the primary sub-types of this cytokine, with VEGF-C and its receptor VEGFR-3 primarily acting to increase mLV lymphangiogenesis and to dilate mLVs. Although early studies demonstrated that VEGF-A and its receptors VEGFR-1 and VEGFR-2 selectively stimulate angiogenesis (generation of new blood vessels), recent studies also indicate a direct lymphangiogenic role of VEGF-A [108].

It should not be surprising that the aforementioned invasive approaches to increase brain VEGF levels via intracerebral injections have only been performed in mice. Along this line, two important mouse studies were published in 2018 that investigated the effects of direct VEGF administration to the brain on mLVs in relation to clearance of brain macromolecules/toxins. Da Mesquite et al. [109] did direct brain VEGF-C treatment of aged mice and reported enhancement in mLV diameter and meningeal lymphatic drainage of CSF macromolecules. Similarly, Wen et al. [110] found that intracerebroventricular infusions of VEGF-C induced lymphangiogenic of mLVs in Alzheimer’s (APP + PS1) transgenic mice. The later mice have the human gene that produces the toxic protein A-beta, widely thought to be the primary Alzheimer’s pathogenic factor upon its self-aggregation into oligomers within the brain. The result of brain VEGF infusions into APP + PS1 mice was a greater clearance of A-beta from their brains as evidenced by reduced soluble A-beta levels in brain/CSF and increased A-beta levels in cervical lymph nodes. These two studies of “brain” VEGF administration in mice clearly demonstrate that there is resulting lymphangiogenesis and increased mLV vessel diameter—both would increase lymph flow through the mLV’s to increase drainage from brain tumors and clearance of brain toxins.

*Animal Studies with VEGF Administration to Induce Glioma Rejection*. In 2020, two landmark papers were published that extended VEGF’s ability for increasing mLV flow to mice bearing glioma cell tumors [105,111]. Both papers demonstrated that brain VEGF treatment to glioblastoma (GB)-bearing mice increased mLV flow to deep cervical lymph nodes to induce a large increase in their generation of memory T cells specific to the glioblastoma. These specific memory T cells then traveled in blood to the brain tumor location, resulting in rejection of the glioma cell tumors. Thus, it has been proposed that *enhancement of the brain’s mLV flow and the resulting specific, robust immune response could be a new therapeutic approach against gliomas and metastatic brain cancers.*

**Figure 7:**
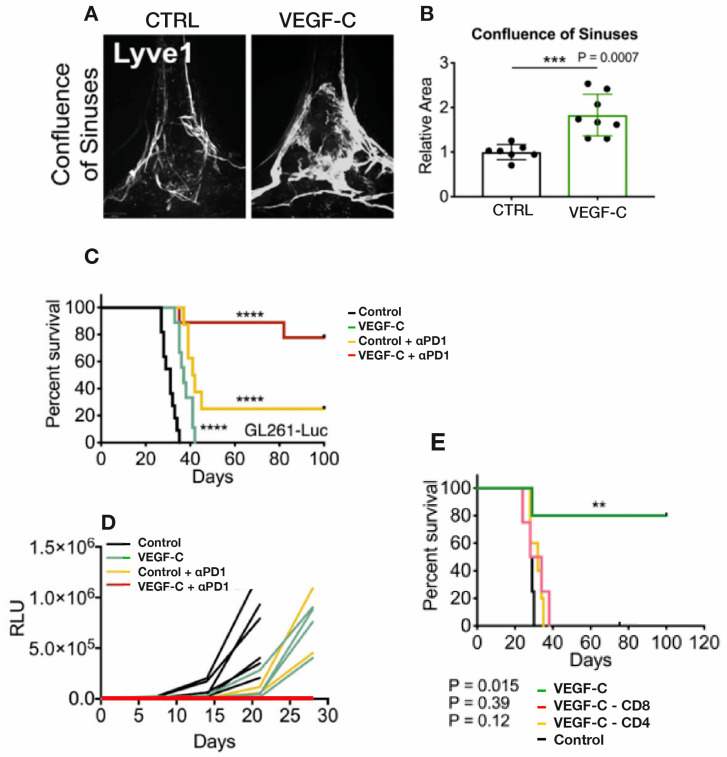
(**A**,**B**) GB tumor-bearing mice received either VEGF or control injection via the cisterna magna. Around 2 months thereafter, imaging of the dura’s lymphatic vasculature revealed a substantial increase in mLV vasculature in the confluence of sinuses induced by VEGF infusion which was quantitatively seen as an increase in mLV area. (**C**,**D**) VEGF- or control-injected mice bearing GB tumors were treated with ɑPD1 antibody or control antibody. Although VEGF injection alone decreased tumor size and increased survival, the combination of VEGF plus ɑPD1 antibody induced complete regression of tumors and a long-term survival of around 80%. (**E**) VEGF intracisternal injection prior to GBM cell injection into mice resulted in long-term survival that was negated by depletion of CD4 or CD8 T cells. Data in (**B**) are presented as the mean ± SD. ** *p* < 0.01; *** *p* < 0.001; **** *p* < 0.0001 (two-way ANOVA). Figures and graphs adapted from Song et al. [105].

In the first of these two 2020 studies involving mice bearing glioblastoma multiforme (GBM) tumors, Song and colleagues [105] found that very limited CD8 T cell immunity to the GB antigen occurs when the tumor was confined to the brain, which resulted in uncontrolled tumor growth. However, following intraventricular VEGF-C mRNA injection in these mice, a clear increase in meningeal lymphatic vasculature was observed in the confluence of sinuses and sagittal sinus (Figure 7A), which was quantitatively underscored by substantial increases in mLV area (Figure 7B). Mechanistically, the increase in mLV flow created by intracerebroventricular injection of VEGF-C mRNA (when accompanied by anti-PD-1 antibody treatment) resulted in substantially increased CD8 T cell presentation/priming to the draining cervical lymph nodes. The resulting enhanced population of CD8 T cells in these nodes then migrated via blood to the tumor, resulting in rapid regression of the GB and a long-lasting antitumor memory response.

Song et al. [105] also found that anti-PD-1 antibody treatment alone had modest effects on survival and tumor size, while the combination of VEGF-C-mRNA with anti-PD-1 antibody resulted in regression of tumors and a long-term survival rate of around 80% (Figure 7C,D). This is consistent with anti-PD-1 and other checkpoint inhibitors showing little or no benefit as a monotherapy in GB clinical trials, which was previously indicated in Section 2. These results strongly suggest that VEGF would have robust synergistic anti-tumor effects when administered with a checkpoint inhibitor. It is important to also note that the enhanced effect of VEGF on survival required lymph drainage to the cervical lymph nodes since ligation of mLVs eliminated VEGF’s survival enhancement. Along this line, depletion of CD4 or CD8 T cells negated the long-term survival protection conferred by VEGF treatment when injected prior to GBM cells (Figure 7E). Mice that had rejected their GB tumors even showed a long-term systemic memory response since re-challenge with GB cells resulted in their complete rejection. The authors conclude that ectopic VEGF-C expression, through enhancement of mLV flow, promotes enhanced CD8 T cell delivery/priming into cervical lymph nodes, robust migration of CD8 T cells into the tumor, and a rapid clearance of the GB. Thus, this eloquent study by Song et al. [105] demonstrated that VEGF treatment can increase the brain’s mLV flow to induce *“a robust and long-lasting T cell-dependent immune response against brain neoplasms”.*

**Figure 8:**
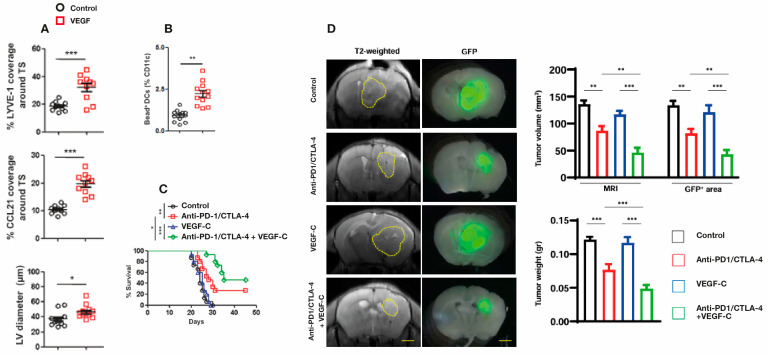
(**A**) Striatal injection of GB cells over-expressing VEGF into mice resulted in an increase in dorsal mLV flow which was quantified by increased mLV coverage/area in both LYVE-1 and CCL21 staining of mLVs, as well as quantitative increases in mLV diameters. (**B**) Quantification of Dendritic cell (DC) trafficking to cervical lymph nodes revealed a significant increase in VEGF-overexpressing mice vs. controls. (**C**) Mice bearing GB tumors that overexpress VEGF-C showed longer survival times after being given anti-PD-1/CTLA-4 compared to GB mice (no VEGF overexpression) given anti-PD-1/CTLA-4 or over-expressing VEGF separately. (**D**) (Left side brain images): representative T2-weighted brain slices and Glia Fibrillary Protein positive (GFP+) stained brain slice areas delineating tumor volumes from mice with (1) intracranial injection of GB cells alone (control), (2) GB cells plus anti-PD-1/CTLA-4 injections, (3) GB overexpressing cells alone, or (4) anti-PD-1/CTLA-4 injections and overexpressing VEGF-C. (Right side bar graphs): quantification of tumor volume and tumor weight in the four aforementioned groups. Dashed lines indicate tumor margin. Scale bars, 3 mm. Data are presented as the mean ± SEM. * *p* < 0.05, ** *p* < 0.01, *** *p* < 0.001; two-way ANOVA. Figures and graphs adapted from Hu et al. [111].

Only one month after Song et al. [105] was published, Hu and colleagues published their mouse work involving injection of GB cells over-expressing VEGF into the striatum [111]. Their findings are complementary in many ways to those reported by Song et al. [105]. Hu et al. [111] found that VEGF over-expression resulted in increased “dorsal” mLV flow, as quantitatively indicated by increased mLV coverage/area and mLV diameters around the transverse sinus for VEGF over-expressing mice vs. controls (Figure 8A). Dendritic cell (DC) trafficking to cervical lymph nodes was then quantified, which revealed much greater DC trafficking in the VEGF group vs. controls (Figure 8B). Both of these results are consistent with a VEGF-induced increase in mLV flow, resulting in enhanced DC trafficking to cervical lymph nodes. Following the administration of anti-PD-1/CTLA-4, mice bearing GBC tumors over expressing VEGF-C exhibited longer survival times than mice given anti-PD-1/CTLA-4 or over-expressing VEGF separately (Figure 8C). Consistent with this survival data, mice bearing tumors overexpressing VEGF-C and given anti-PD-1/CTLA-4 had decreased tumor volumes and tumor weight compared to control, anti-PD-1/CTLA-4, or VEGF-overexpression groups alone (Figure 8D). These profoundly beneficial effects of VEGF-C overexpression on dorsal mLV flow were all abolished in mLV-defective mice, indicating a significant role of dorsal mLVs in brain tumor drainage and immune-based tumor rejection [111]. It had previously been reported [112,113] that the combination of anti-PD-1/CTLA and VEGF activates T cells in both the tumor microenvironment and draining cervical lymph nodes (CLNs), both of which appear to have contributed to the antitumor response observed by Hu et al. [111]. Moreover, results from Hu et al. [111] advocate that increased DC trafficking to CLNs under enhanced meningeal lymphangiogenesis by VEGF elicits stronger immune responses against brain tumors. Both Song et al. [105] and Hu et al. [111] underscore that enhancement of mLV flow is critical for generating a robust and efficient T-cell dependent immune response against brain tumors.

It is important to mention the angiogenic ability of VEGF and use of anti-VEGF drugs in brain cancer clinical trials. These trials posited that anti-VEGF drugs (e.g., Bevacizumab) would induce tumor regression by decreasing brain tumor vascularity [1] (see Section under Immune Therapeutics). As previously indicated though, anti-VEGF drugs (e.g., VEGF Traps such as Ziv-aflibercept, Bevacizumab) have been unsuccessful in treating gliomas. Indeed, use of anti-VEGF drugs against brain tumors would appear to be exactly opposite to this perspective paper’s proposed therapeutic actions of TRFT involving VEGF. However, VEGF has in fact been shown to enhance (not suppress) the response to immunotherapy in mouse melanoma cells [114]. The authors proposed that VEGF-C potentiates immunotherapy by attracting naïve T cells to the tumor, which are then locally activated with immunotherapy-induced killing of tumor cells. In another study involving GB patients treated with anti-PD-1 [105], a high correlation between VEGF-C within the tumor and increased tumor T cell infiltration (CD3e, CD4, CD8B) was shown after treatment, suggesting that the GB microenvironment is normally deprived of lymphangiogenic signals. As such, the level of VEGF in both CSF/mLVs and in the brain tumors themselves appears to be more important than plasma VEGF levels for determining the benefits of VEGF for induction of tumor regression.

*Intracerebral VEGF administration to treat human brain cancers is invasive, risky, and impractical*. The aforementioned pre-clinical work in mice is very encouraging for modulation of brain VEGF levels and thus modulation of mLVs to induce brain tumor regression. However, it is important to underscore that direct intracerebral injection of VEGF or VEGF vectors into the brain’s CSF via the cisterna magna or into the brain’s cerebral ventricles has thus far only been done in rodents. Moreover, direct administration of VEGF into the human brain’s CSF to treat brain cancers would be invasive (require initial surgery), risky (infection/dose-related issues), and inconvenient (require continual clinical visits for brain infusions) Continuously administered VEGF is another clinical possibility, but the viability and effective concentration of long-term VEGF infusion solutions is unknown in humans and mouse studies have not been performed yet. As such, other interventions are clearly needed clinically that are both non-invasive and effective long-term in modulating brain/CSF levels of VEGF to treat brain cancers. This paper forwards Transcranial Radiofrequency Wave Treatment (TRFT) as fulfilling both of these requirements. The clinical evidence supportive of these TRFT actions are presented in the next Section.

## 5. Clinical Effects of TRFT on VEGF, Brain Toxin Drainage, and Likely Brain Lymphatics

As underscored in the previous section involving mice, VEGF-induced enhancement of brain lymphatic flow via meningeal lymphatic vessels (mLVs) is critical for generating a specific and robust T-cell dependent immune response to brain tumors. [105,111]. In humans, the only known way to non-invasively modulate brain VEGF levels is through TRFT. As such, this section will present clinical data from AD patients demonstrating how TRFT can increase VEGF levels in both brain and blood to ostensibly increase lymphatic drainage of toxins (e.g., A-beta1-42, p-tau, t-tau) from the brain. Such increased mLV flow should in the same way present cervical lymph nodes with greater T-cell and DC immune cell numbers from brain tumors, resulting in a more vigorous cervical lymph node-based immune attack on brain tumors via the blood.

**Figure 9:**
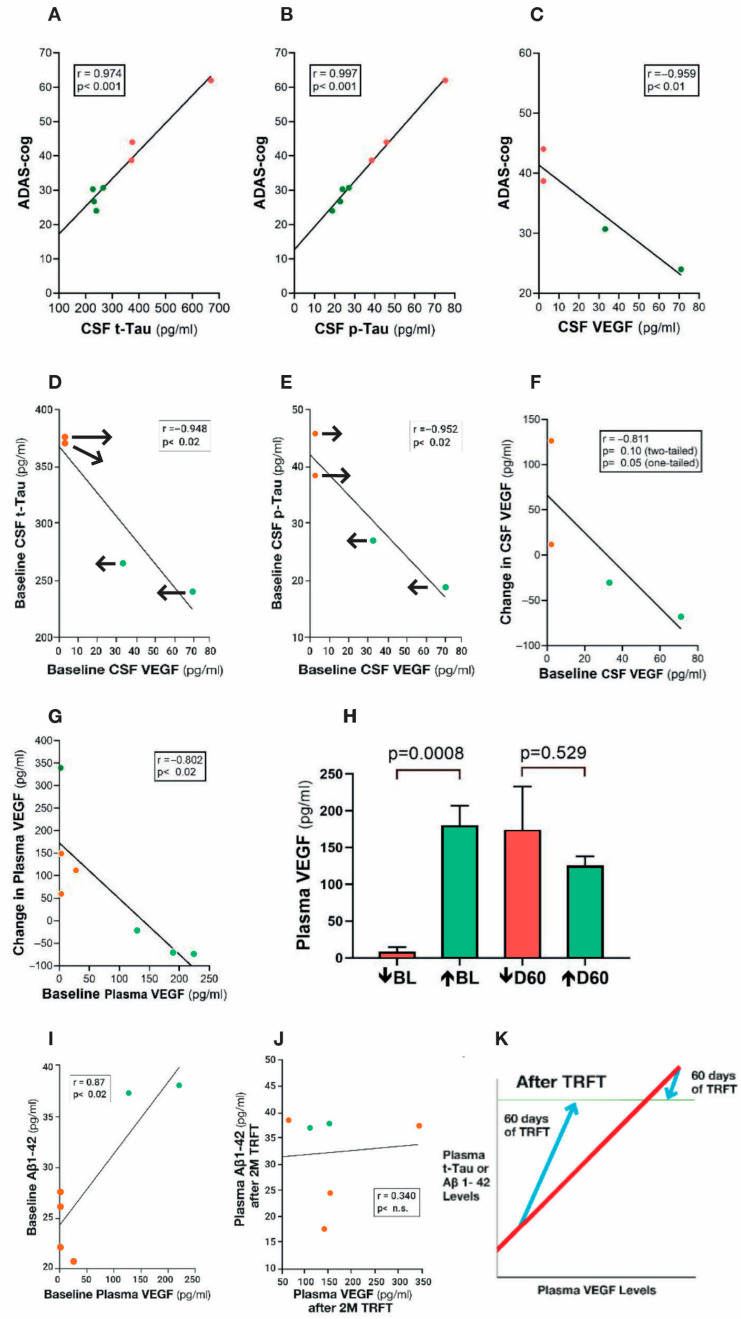
(**A**–**C**) ADAS-cog scores strongly correlate with CSF levels of t-tau, p-tau, and VEGF. Higher levels of t-tau and p-tau correlate with poorer ADAS-cog performance, while higher levels of VEGF correlated with better ADAS-cog performance. Red dots represent AD subjects with higher (poorer) ADAS-cog scores, while green dots represent AD subjects with lower (better) ADAS-cog scores. (**D**,**E**) CSF t-tau and p-tau levels are negatively correlated with CSF levels of VEGF: TRFT re-balances these AD markers to eliminate their correlations with VEGF (arrows). TRFT re-balances VEGF levels in both CSF (**F**) and plasma (**G**). If baseline VEGF levels were low, TRFT increased those levels (red circles) and vice versa if VEGF levels were high (green circles). This re-balancing of VEGF by TRFT is also evident in bar graph format for plasma (**H**), wherein AD subjects were divided into two groups—low or high baseline (BL) plasma VEGF levels. The large difference in plasma VEGF between these two groups at BL was eliminated by 2 months of TRFT. (**I**) Baseline plasma Aβ 1-42 levels are directly correlated with plasma VEGF levels. (**J**) TRFT re-balances Aβ1-42 to eliminate this correlation with VEGF. Subjects with low baseline (BL) levels of VEGF are indicated by red circles, while those with high BL VEGF levels are indicated by green circles. It is evident that this re-balancing by TRFT primarily involved an increase in AD marker levels in subjects with low BL levels of VEGF. (**K**) As summarized in this graph, the re-balancing of AD markers in plasma by TRFT (as exemplified in (**I**,**J**)) primarily involves an increase in AD marker levels in subjects with low BL levels of VEGF to eliminate correlations. Figure 9A–J adapted from Arendash et al. [115].

Clinical evidence for VEGF being critical for toxin clearance from the brain and better cognitive function can be gleaned from the strong correlations present between cognitive function (ADAS-cog performance) and CSF levels of t-tau, p-tau, and VEGF in AD subjects at baseline [115] (Figure 9A–C). Subjects could be divided into two groups based on their cognitive performance. Poorer baseline cognitive performance (higher ADAS-cog score) and higher t-tau and p-tau levels are seen in AD subjects with low VEGF levels, while better baseline cognitive performance and lower t-tau and p-tau levels are present in AD subjects with high VEGF levels. These two contrasting associations would appear to involve VEGF’s direct ability to increase mLV flow, resulting in greater brain clearance of toxins and better cognitive performance.

When AD subjects are divided into two groups based on lower or higher baseline VEGF levels in CSF/brain, higher levels of baseline VEGF in CSF/brain were associated with enhanced drainage of t-tau (295% higher) and Aβ1-42 (48% higher) into plasma compared to the low VEGF group [115]. Moreover, there were robust negative correlations in CSF (mLVs) between baseline levels of VEGF and baseline CSF levels of both t-tau and p-tau **(**Figure 9D,E)—higher levels of VEGF in CSF (mLVs) were associated with an increased clearance of these AD markers from the brain/CSF and into plasma. Administration of daily TRFT for 2 months eliminated these correlations by increasing CSF VEGF levels in subjects with low baseline VEGF levels and decreasing CSF levels in subjects with higher baseline VEGF levels (Figure 9D,E; arrows). These TRFT-induced changes in CSF t-tau and p-tau levels were clearly linked to TRFT-induced changes in CSF VEGF levels, with TRFT reducing higher baseline VEGF levels and increasing lower baseline VEGF levels (Figure 9F). Thus, VEGF levels in CSF/brain were “re-balanced” by TRFT, as were CSF levels of both t-tau and p-tau. The mechanism for this re-balancing of VEGF in CSF/brain by TRFT likely involves modulation of one, two, or all three sources of VEGF in the brain: namely, plasma in choroid plexus capillaries, tissue macrophages, and choroid plexus ependymal cells (Figure 6).

The same re-balancing of VEGF by TRFT was also present in plasma (Figure 9G), as also depicted in bar graph format dividing subjects into those with low and those with high VEGF levels at baseline compared to normal VEGF levels of about 100 pg/mL [116] (Figure 9H). TRFT re-balanced plasma VEGF levels by increasing levels in the low baseline group and modestly decreasing levels in the higher baseline group—the result was no “low” versus “high” group difference after 2 months of TRFT. At baseline, three AD markers in plasma (t-tau, Aβ1-40, and Aβ1-42) were directly correlated with plasma VEGF levels (example of Aβ1-42 correlation shown in Figure 9I). These correlations suggest that higher levels of plasma VEGF are resulting in increased clearance of all three AD markers from brain/CSF into plasma. However, daily TRFT for 2 months eliminated all three of these three correlations—Figure 9J shows the example of Aβ1-42. It should be noted that only four of the seven subjects had measurable brain levels of VEGF-A, which was evaluated rather than the much more prevalent VEGF-C (30:1 ratio). Both VEGF sub-types parallel one another in concentration, with the VEGF-A sub-type not only inducing lymphangiogenesis [117], but also stimulating release of VEGF-C from resident macrophages [118]. As previously indicated, VEGF-C induces lymphangiogenesis as well as evokes dilation of lymphatic vessels [108,110].

Figure 9K summarizes the effect of TRFT on plasma AD markers by showing the correlations with VEGF were eliminated by (1) robust TRFT-induced increases in plasma AD markers for those AD subjects with low baseline VEGF levels, and (2) small or no TRFT-induced decreases in AD markers for those AD subjects with high baseline levels. The mechanism(s) for TRFT-induced re-balancing of VEGF levels in blood will be presented in Section 8. It can be indicated now, however, that there are likely two different mechanisms of TRFT action in CSF/brain vs. plasma to regulate VEGF levels because there was no correlation between VEGF levels in these two compartments, as was previously reported [119].

***Conclusions.*** The AD marker changes (re-balancing) in plasma and CSF shown clinically after 2 months of TRFT would appear to be primarily due to VEGF’s modulation of mLV flow and associated modulation of brain toxin removal. Particularly in subjects with “low” baseline VEGF levels in CSF/Brain (or plasma), a TRFT-induced increase in VEGF levels would target mLVs to increase mLV dilation and lymphangiogenesis—both of which would increase mLV flow and toxin removal from the brain into venous blood. ***In brain cancer patients, this same increase in mLV flow by TRFT could result in a greater lymphatic flow of immune-specific T-cells and dendritic cells from the tumor to cervical lymph nodes and a resultant more robust immune response into blood back to the tumor, resulting in tumor regression.***

## 6. A Proposed Mechanism of TRFT Action Against Brain Tumors: Modulation of Low VEGF Levels to Increase Brain Lymphatic Flow

The initial step for TRFT to treat brain cancers should be determining the blood and CSF/brain levels of both VEGF-A and VEGF-C. Low or undetectable plasma VEGF-A levels (compared to normal levels of around 100 pg/mL) [116] are often seen in “low-grade” gliomas [120]. Patients bearing such low-grade gliomas would be good candidates for TRFT-induced increases in VEGF in brain/CSF. That would cause dilation of mLVs (primarily dorsal), resulting in increased lymph flow and the cascade of immune-activating events shown in Figure 5 to facilitate transport of memory T-cells and dendritic cells from the tumor to cervical lymph nodes. At cervical nodes, a resulting enhanced and aggressive immune response of tumor-specific T-cells and DCs is produced that feeds back directly to the brain tumor via the blood. Thus, TEMT-induced VEGF alone, or perhaps in combination with standard glioma treatments (e.g., anti-PD-1/CTLA-4), could result in immune-induced glioma regression and the patient’s remission, as was seen with VEGF enhancement in glioblastoma-bearing mice [105,111].

In contrast to low-grade gliomas, “high-grade” glioma patients usually have elevated VEGF levels in their CSF/brain [119,120,121], within their glioma [122], and in their plasma [123]. As such, TRFT should modestly reduce these high levels without appreciably affecting VEGF regulation of mLV flow, as was shown to be the case in AD subjects (see preceding section). Irrespective of whether VEGF levels in the CSF/brain and/or plasma of glioma subjects are high or low, clinical studies have shown that pro-inflammatory cytokines (e.g., IL-1, IL-6, IL-17, IL-18, TNF-ɑ), anti-inflammatory cytokines (e.g., IL-4, IL-6, IL-10, IL-13), and growth factor cytokines (GCSF, GMCSF, TGF-β) are also out-of-balance in being too high or too low—the result is out-of-control inflammation [121,123]. As such, it is critical to re-balance ALL of these cytokines (not just VEGF) to substantially reduce inflammation in the TME and CSF/brain. Thus, a second therapeutic target of TRFT against all brain cancers is to address the cytokine imbalance and resulting inflammation within the brain tumor and brain—specifically, to re-establish a healthy balance between pro- and anti-inflammatory cytokines. Such an environment within the brain tumor and surrounding brain should result in much reduced inflammation therein and an ensuing inhospitable, unsupportive environment for tumor viability—this should then bring about tumor regression or elimination.

The next section discusses cytokine imbalance and inflammation in and around the microenvironment of brain tumors and the clinical evidence for the tumor microenvironment and surrounding brain tissue being a second therapeutic target for TRFT.

## 7. A Second Target of TRFT Against Brain Cancers: Inflammation/Cytokine Imbalance Within the Brain Tumor and Brain in General

**Figure 10:**
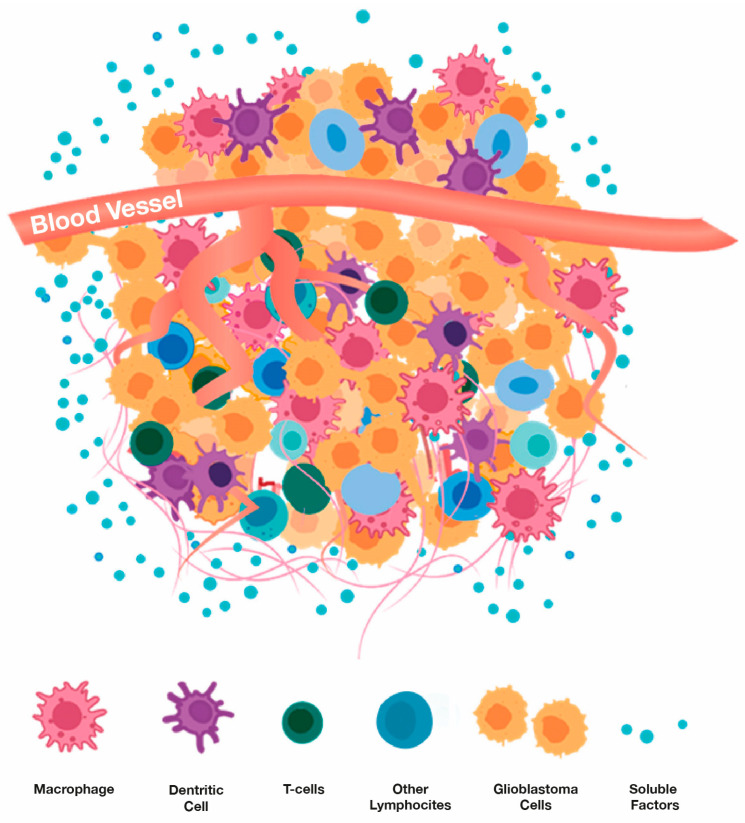
Cell types and soluble factors (e.g., cytokines, growth factors) that comprise a glioblastoma’s microenvironment. Adapted from Li et al. [142].

The tumor microenvironment (TME) of glioblastomas is heterogeneous in consisting of both non-cellular and cellular components [124,125,126,127,128,129,130] (Figure 10). The non-cellular components include various soluble factors (e.g., growth factors, cytokines, nutrients, metabolic wastes). The cellular components are composed of glioma cancer cells and inflammatory cells, with inflammatory cells making up 30–50% of the tumor mass. Inflammatory cells interact with cancer cells and consist primarily of macrophages, T cells, and dendritic cells. **Macrophages** are collectively referred to as tumor-associated macrophages (TAMs) and are far and away the predominant inflammatory cell populations in glioblastomas [131]. Circulating macrophages in blood are attracted to the cytokine milieu within the TME and secrete cytokines themselves once within the TME. These secreted cytokines obstruct the function of T cells within the TME [132]. **T-cells** are present in the TME in two sub-populations. CD4+ T cells regulate the overall TME in an immunosuppressive manner [133,134]. This then inhibits production of anti-tumor cytokines. The other sub-population, CD8+ cytotoxic T cells, induces a tumor-killing effect and mediates tumor regression. An apparent reason why immune checkpoint Inhibitors (e.g., PD-1 inhibitor) have not been more efficacious against glioblastomas is the scarcity of infiltrating CD8+ T cells into the TME [135]. Finally, another important cell type in the TME are **Dendritic cells** (DCs), which produce anti-tumor cytokines, to in turn attract more CD8+ T cells to the TME [136].

Glioblastomas are characterized as having chronic inflammation, not only within the tumor/TME, but also in the CSF/brain tissue and blood of the brain cancer patient. This inflammation is consistently identified as cytokine levels being abnormal. **Within gliomas** themselves, for example, VEGF is secreted mainly by tumor cells [137], with VEGF levels in glioma supernatant being higher compared to that of very slow-growing glioma controls [138]. In glioma cell cultures derived from various human glioma cell lines, consistently robust levels of G-CSF and its receptor were found [139]. As well, high levels of GM-CSF were found exclusively within the highest-grade gliomas [140]. In two other studies, both VEGF and IL-6 levels within human gliomas were positively associated with tumor grade [122,141]. These studies suggest that progressive increases in GM-CSF, VEGF, and IL-6 levels within the TME play an important role in the evolution and pathogenesis of gliomas.

Turning to **CSF/brain** cytokine levels, CSF levels of the cytokines IL-2, IL-6, and VEGF from glioblastoma subjects (Stage III and IV) were found to be significantly elevated compared to control subjects, while IL-7 levels were significantly reduced [119,120,121,143]. In other studies, increased levels of IL-6 in the CSF have been reported and associated with tumor infiltrating macrophages in gliomas/glioblastomas [144,145]. Even the **blood** of glioma subjects has abnormal cytokine levels, as evidence by serum GCSF levels being 53% higher in glioma patients compared with healthy controls [146]. Along that line, a complex dysregulation in serum levels of multiple cytokines was reported for glioblastoma subjects compared to controls [123]. In that study, 2- to 3-fold higher levels of the pro-inflammatory cytokines involved in tumor progression and aggressiveness (IL-6, IL-17, IL-18, and TNF-ɑ) were found in serum from glioblastoma subjects vs. controls. Similar increases were seen in serum levels of the anti-inflammatory cytokine IL-10 and in the growth factor cytokines VEGF and GM-CSF, while significant decreases were reported in levels of the anti-inflammatory cytokines IL-4 and IL-12.

Clearly, the heterogeneous characteristics of the glioblastoma microenvironment (Figure 10) leads to an imbalance in cytokines within the tumor/TME, as well as in the CSF/brain in general and the blood. As such, glioblastomas induce a dysregulated immune response resulting in a state of chronic inflammation involving both pro- and anti-inflammatory cytokines, as well as various growth factors. Such immune chaos favors tumor progression and resistance to anti-inflammatory therapies. A therapeutic intervention is needed to provide an overall re-balancing of immune function in the tumor-bearing brain; this, to make for an inhospitable environment for tumor growth/survival within the tumor’s brain environment. The only intervention that has been clinically shown to re-balance brain (and body) cytokines to reduce brain (and body) inflammation is TRFT [104,147]. The next section provides the clinical evidence for this re-balancing, which should result in brain tumor rejection and the patient’s long-term survival.

## 8. A Second Mechanism of TRFT Action Against Brain Tumors: Re-Balance the Immune System to Decrease Inflammation in Both Brain and Blood

The first mechanism of TRFT action against brain tumors involved modulation of VEGF levels in the brain and blood to affect brain lymphatic (mLV) flow. If TRFT increases mLV flow, a greater immune stimulus would arrive at cervical lymph nodes resulting in a greater immune presentation to the brain tumor. However, irrespective of whether VEGF levels in the CSF and/or plasma of glioma subjects are high or low, all primary and metastatic brain tumors are characterized by an out-of-balance cytokine system manifesting itself within the TME, brain, and blood as chronic inflammation that nurtures tumor development [121,123,142]. This section provides the clinical evidence that TRFT can re-balance many cytokines in both CSF/brain and blood of Alzheimer’s subjects to greatly reduce inflammation in both their brain and blood. It is proposed that the same cytokine re-balancing and reduction in inflammation will occur for brain cancer subjects to induce tumor rejection.

**Figure 11:**
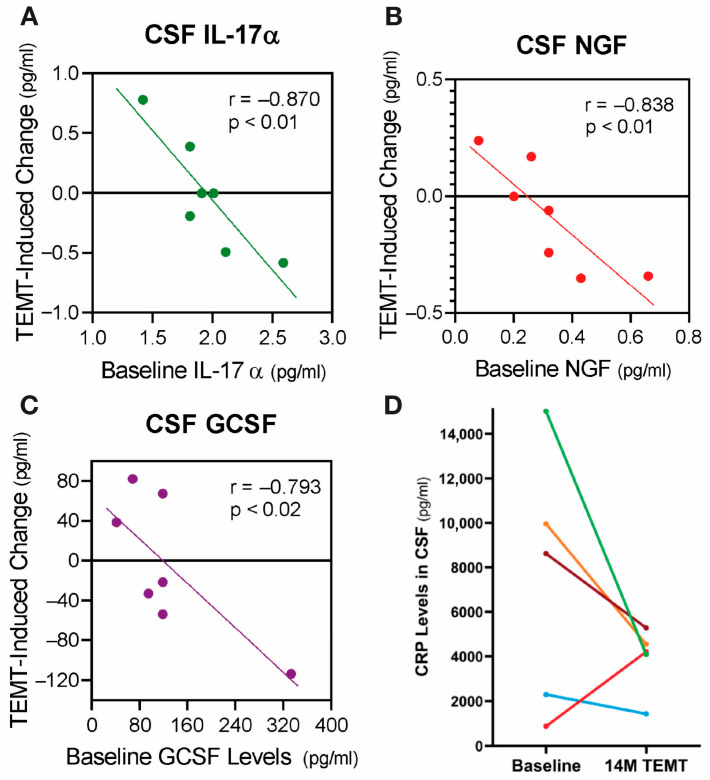
(**A**–**C**) Three examples of the significant correlations resulting from the rebalancing of CSF cytokine levels by TRFT in individual AD subjects (colored dots). If baseline CSF levels were low (below the horizontal “convergence” dashed line), TRFT resulted in increased blood levels after 2 months of treatment. Just the opposite occurred if baseline levels were high. Colored lines are best fit lines with correlation analysis. (**D**) For individual AD subjects, effects of 14 months of TRFT on C-reactive protein (CRP) levels in CSF also indicate a rebalancing by TRFT—higher baseline CRP levels (4 subjects) resulted in an overall lowering by 51% and very low baseline CRP levels (1 subject) resulted in an increase. Figure 11A–C adapted from Cao et al. [147] and Figure 11D adapted from Arendash et al. [104].

Several studies have investigated effects of full-brain TRFT (via MemorEM devices) on cytokine and inflammatory marker levels in CSF/brain of Alzheimer’s subjects. In an initial study, seven AD subjects were treated daily in-home with TRFT by their caregivers for 2 months [147]. For all seven cytokines measurable in CSF (IL-8, IL15, IL-17ɑ, VEGF, NGF, GCSF, and TGF), the response to TRFT was dependent on baseline CSF levels. If a low level of any given CSF cytokine was present at baseline, 2 months of TRFT increased those levels; if a higher level was present at baseline, 2 months of TRFT decreased those levels. Three examples of this cytokine re-balancing in CSF by TRFT are shown in Figure 11A–C. Thus, a 2-month period of daily TRFT can re-balance all seven measurable cytokines in CSF.

C-Reactive Protein (CRP) is a protein that is a widely accepted measure of general inflammation, with brain levels of CRP increasing when a condition is causing brain inflammation. In a second study involving inflammation in AD subjects [104], CSF levels of CRP were taken at baseline and at 14 months into TRFT. Compared to baseline, TRFT reduced CSF/brain levels of CRP in four of the five subjects at 14 months by an average of 51% (Figure 11D). The one subject who showed a TRFT-induced increase in CRP levels had very low baseline levels, indicating that a re-balancing of CRP had occurred. Thus, TRFT not only re-balanced CSF/brain cytokines, but the consequence of that appear to be a large decrease in brain inflammation over an extended period. Indeed, this re-balancing of cytokines and decreased brain inflammation could be responsible for the long-term cognitive stability of 2½ years provided to these AD subjects by TRFT [104]. ***The same above re-balancing of brain cytokines and ensuing decrease in brain inflammation could occur with TRFT to brain cancer subjects—the result being an environment inhospitable to tumor viability and consequent tumor regression.***

The mechanism(s) of TRFT action on brain cytokines is currently unknown. In the case of brains bearing primary or metastatic tumors, the TME is well known to secrete high amounts of multiple cytokines [122,140,141] resulting in inflammation within the TME, as previously discussed. Although TRFT is certainly reaching all cells within the TME, the extent to which TRFT re-balances cytokines within the TME is an important future question.

**Figure 12:**
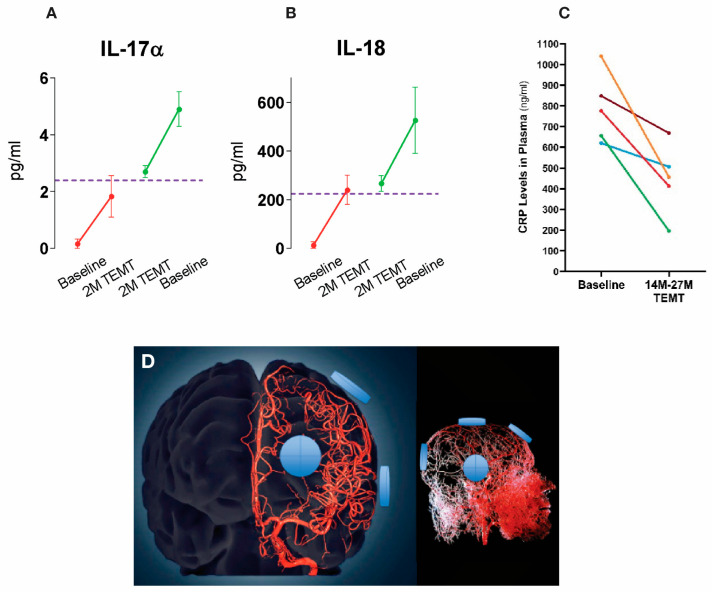
(**A**,**B**) Levels of two pro-inflammatory cytokines in plasma at baseline and after two months of daily TRFT (means ± SEMs). For both cytokines, the response to TRFT is dependent on baseline levels, with TRFT inducing convergence (rebalancing) of both IL-17 and IL-18 toward normal levels (horizontal dashed line). (**C**) The effects of 14–27 months of TRFT on plasma CRP levels in individual subjects showing an overall TRFT-induced decrease of 43%. (**D**) A three-dimensional mode of the cerebral arterial tree showing the location of four MemorEM device RF emitters on the head’s surface on the right side. The emitters (blue disks) are in close proximity to affect all blood components within the cerebrovascular tree including arteries/arterioles and most importantly capillaries, where the velocity of blood cells and plasma is at its slowest for maximal radiofrequency wave exposure. Graphs in A and B adapted from Cao et al. [147]. Graph in “C” adapted from Arendash et al. [104]. Figure in “D” adapted from Arendash et al. [102].

In the same AD subjects wherein TRFT re-balanced seven “CSF/brain” cytokines, TRFT also re-balanced 11 or 12 cytokines in “plasma” (GCSF, GMCSF, VEGF, PDGF, IL-8, IL-10, IL-15, IL-17ɑ, IL-18, TGF-ɑ, and IFN-γ [147]. Two examples of this cytokine re-balancing in plasma by TRFT are shown in Figure 12A,B for the pro-inflammatory cytokines IL-17ɑ and IL-18. As was the case for previously discussed re-balancing of VEGF in both plasma and brain, AD subjects with lower-than-normal baseline levels of a particular cytokine in plasma showed an increase in that cytokine after 2 months of daily TRFT. However, those AD subjects with higher-than-normal baseline levels of a given cytokine in plasma displayed a decrease in that cytokine after 2 months of TRFT. Subjects with baseline levels for a given cytokine that were very near normal levels showed little or no change following TRFT.

The re-balancing of so many plasma cytokines (11 of 12) by TRFT was apparently responsible for the 43% reduction in plasma CRP levels (788 75 ng/mL vs. 448 76; *p* = 0.017) for five of these AD subjects after an extended 14-month treatment period (Figure 12C) [104]. The mechanism for these dramatic TRFT effects to re-balance plasma cytokines and reduce plasma inflammation likely involves direct effects of RF waves on red blood cells (RBCs) in blood flowing through the head and brain, underneath the eight RF emitters (Figure 4A and Figure 12D). RBCs highly concentrate cytokines, resulting in RBC concentration gradients with plasma that normally average 12:1 for 46 cytokines [148]. RF wave exposure of human RBCs going through the head/brain vasculature causes increased RBC permeability that increases blood cytokines in or out of RBCs depending on their plasma cytokine concentration [102]. As such, if a given cytokine’s concentration in plasma is lower than normal, a net flux of that cytokine across RBC membranes and out of RBCs occurs, with just the opposite occurring if plasma cytokine levels are too high. Since TRFT’s modulation of the cytokine/inflammatory environment in “brain/TME” would appear to be most relevant to tumor regression, these modulatory effects of TRFT on plasma cytokines/inflammation are presently of unknown importance.

It is noteworthy that in the same AD subjects wherein cytokines in both CSF/brain and blood were re-balanced by 2 months of TRFT, a reversal of AD cognitive impairment was also observed [103]. It is reasonable to expect that a similar TRFT-induced re-balancing of cytokines will occur in the brain and TME of brain cancer patients, resulting in decreased brain inflammation, tumor regression, and long-term survival.

## 9. A Third Target of TRFT: Possible Direct Actions on Brain Cancer Cells

TRFT has already been described to have two actions that should attack both primary and metastatic brain cancers: (1) modulation of brain VEGF levels to presumably increase brain lymphatic flow for an enhanced immune attack on brain tumors; (2) re-balancing of brain cytokines to decrease brain inflammation, not only in the brain in general, but hopefully within the TME. The third “prong” of TRFT’s 3-pronged attack on brain tumors involves possible direct actions on tumor cells themselves within the TME.

All of the evidence for direct actions of RF waves on brain cancers has involved in vitro cell culture studies (i.e., no blood or lymphatic circulation and no surrounding neuronal/glia environment). Although such studies do not closely mimic the TME of animals or humans, they do provide some insight into RF wave effects directly on various tumor cell lines. An early study investigated glioblastoma and neuroglioma cells exposed to 2.15 GHz RF waves and found no effects on cancer cell growth or viability [149]. However, three later studies all reported inhibitory effects of RF waves on various brain call cell lines. Exposure of U-87 MG human glioblastoma cells to RF frequencies between 100 mHz and 3.5 GHz for 54 h resulted in a significant 40% reduction in glioblastoma cell numbers [150]. Thereafter, SH-SY5Y neuroblastoma cells were significantly decreased in growth rate and proliferation by 1760 MHz RF waves after four days of 4 h/day treatment [151]. Finally, the administration of 2.4 GHz RF waves to U-118 MG glioblastoma cells for 24–72 h resulted in a significant decrease in viability [152]. These three studies share the commonality of RF waves directly compromising the viability of brain cancer cells. Consequently, TRFT may effectively target the cancer cells that comprising the majority of cells within a given brain tumor’s TME, especially with its ability to permeable all areas and cells of the human brain.

It is important to distinguish between the presently utilized “RF waves” within the electromagnetic spectrum and other very different neuromodulatory technologies that involve magnetic stimulation (e.g., Pulsed Electromagnetic Stimulation; PEMF) or alternating electric fields (e.g., Tumor Treating Fields; TTFs)—neither of these technologies involve true “electromagnetic” waves (Figure 2A). In being very different from the “electromagnetic” RF waves utilized in all of the aforementioned brain cell culture studies, TTFs have been approved by FDA for treatment of glioblastomas (with or without adjuvant TMZ). The result is a reported reduction in proliferation and migration of glioma cells induced by TTFs, which occurs in a frequency- and power level-dependent fashion [153]. However, the early glioblastoma cell culture studies of Kirson et al. [154] reporting cell death with “clinically-utilized” low intensity (1–3 V/m) electric fields have not been duplicated in more recent studies involving multiple glioblastoma cell lines [155].

Thus, the efficacy of TTFs to clinically treat glioblastomas is now being questioned by some. It seems more tenable that TRFT can induce “direct” anti-proliferation/antigrowth affects in brain tumors because: (1) multiple studies and investigators have shown RF waves to have such effects in multiple brain cancer lines, and (2) TRFT’s RF waves can generate an electric field within the human brain that is much stronger and goes deeper than that of TTFs (40 V/m vs. 1–3 V/m) [156]. Additional glioma cell culture studies are needed to more firmly establish the ability of TRFT/RF waves to inhibit cell proliferation within gliomas or other types of brain cancer cells.

## 10. Advantages and Limitation of TRFT Against Brain Cancers

**Figure 13:**
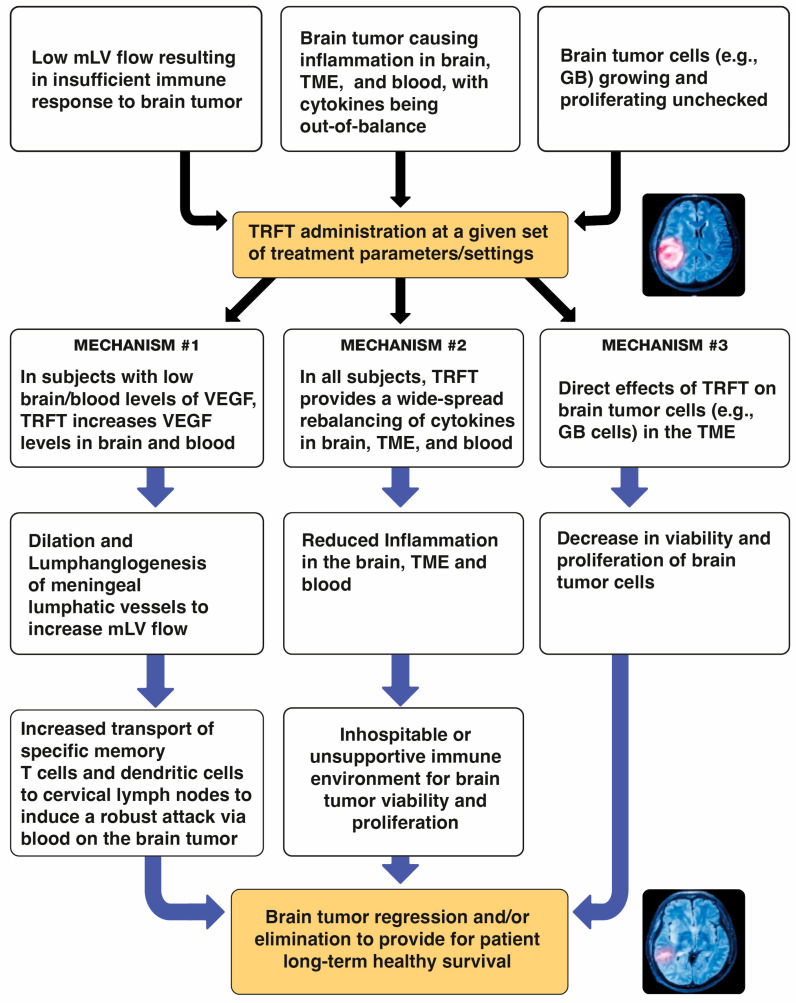
Summary of the 3 targets for TRFT and 3 possible mechanisms of action for TRFT against brain cancers. TME = tumor microenvironment.

**Advantages:** A huge potential advance of TRFT is that it offers three viable mechanisms of action against brain cancers—not just the singular mechanism that most drugs can offer (Figure 13). With the possible exception of TTFs, TRFT would appear to be the only non-pharmacologic intervention against brain cancers to provide complete brain treatment for eliminating even undetected cancer cells in the brain. Relatedly is TRFT’s ability to penetrate all cells of the human brain, thus negating all blood–brain barrier and brain-tumor barrier issues. And unlike RF thermal ablation or other interventions targeting cancer cells in the brain, TRFT’s actions may kill cancer cells without denaturing their released proteins, thus inducing a more robust/specific immune response by memory T-cells/dendritic cells. In contrast to TRFT, none of the current therapeutic interventions against brain cancer appear capable of re-balancing the human brain’s immune system, resulting in decreased brain inflammation. Moreover, none involve activation of the immune system through enhanced lymphatic flow through meningeal vessels and most, if not all, have significant deleterious side effects. Most importantly, *TRFT theoretically should effectively treat all forms of primary and metastatic brain cancer*—*and with no or minimal detrimental effects on cognition or other brain functions.*

**Current Limitations:** Despite the many apparent advantages listed above for TRFT to safely and effectively treat brain cancers, a number of limitations currently exist that can only be addressed through clinical administration of TRFT to both primary and metastatic brain cancer patients. Some of these limitations are listed below:

(1) No TRFT studies in either primary or metastatic brain cancer have been performed yet. The currently presented evidence supportive that TRFT could have benefits against brain cancers is derived from a diverse array of studies involving both glioblastoma cell/animal models and clinical trials in Alzheimer’s Disease subjects.
(2) Although a dozen cytokines and a major marker of inflammation (CRP) have been evaluated in the CSF/brain of humans and shown to be modulated by TRFT, these immune markers have not been evaluated for TRFT modulation in brain cancer patients and measurement of a wider array of immune markers would be most desirable. Moreover, the ability of TRFT to modulate any type of immune cell (e.g., macrophages, T cells, dendritic cells) in human CSF or blood has not been determined.
(3) Although TRFT as a monotherapy against brain cancers is conceivable, it is likely that TRFT will require adjuvant drug treatment (e.g., with a PD-1 inhibitor) to be most effective.
(4) As with any new and pioneering treatment in medicine, neuro-oncologists may be reluctant to try a neuromodulatory-based intervention that they have probably not heard of or know nothing about until there is compelling clinical evidence of its efficacy against brain cancers.

Nonetheless, many clinical trials of drugs in brain cancer subjects have been initiated with less supportive data than is presently being forwarded. Indeed, the current huge barriers of the BBB and BTB for sufficient delivery of drug therapeutics to brain tumor location(s) substantially lessens their ability to provide long-term benefits—yet the BBB and BTB are easily traversed by the non-thermal radio waves of TRFT technology.

## 11. Immediate TRFT Clinical Trials to Treat Brain Cancers Are Now Justified

Collectively, the cell culture studies, glioma mouse studies, and TRFT clinical studies presented in this perspectives paper provide an exciting and hopeful argument for clinical trials of this bioengineered technology to be initiated now in brain cancer patients. TRFT technology is safe, non-invasive, and easily administered at home. As shown in Figure 13, its three mechanisms of action (targets) against brain cancers provide a greater opportunity for success compared to current ineffective drugs that can generally offer only one mechanism of action (target). Indeed, TRFT represents an entirely new *modus operandi* to potentially treat all types of brain tumors, both seen and unseen—especially primary brain cancers such as gliomas, wherein the majority of therapeutic interventions have been tested thus far. Thus, it is concluded that the pre-clinical and clinical research presented in this perspectives paper warrant clinical trials of TRFT (either as a monotherapy or in combination with drug therapies) in both primary and metastatic brain cancer patients. Certainly, they deserve this chance for long-term “healthy” survival.

## 12. Patents

The treatment of primary and metastatic brain cancers by Transcranial Radiofrequency wave treatment (TRFT; TEMT) is protected by claims in U.S. Patent #11,911,629 B2 (G. Arendash, Inventor). The potential capacity of TRFT to increase mLV flow through enhancement of brain/blood VEGF levels is protected by claims in U.S. Patent #11759650-B2 (G. Arendash and R. Baranowski, Inventors).
